# Promoting work ability with a wearable activity tracker in working age individuals with hip and/or knee osteoarthritis: a randomized controlled trial

**DOI:** 10.1186/s12891-022-05041-1

**Published:** 2022-02-03

**Authors:** Elin Östlind, Frida Eek, Kjerstin Stigmar, Anita Sant’Anna, Eva Ekvall Hansson

**Affiliations:** 1 Department of Health Sciences, Division of Physiotherapy, Lund University, Lund, Sweden; 2 Dalby Healthcare Center, Region Skåne, Lund, Sweden; 3 Skåne University Hospital, Lund, Sweden; 4 Viniam Consulting AB, Halmstad, Sweden

**Keywords:** Hip osteoarthritis, Knee osteoarthritis, Physical activity, Wearable activity tracker, Work ability, Mhealth

## Abstract

**Background:**

Physical activity (PA) may improve work ability and health in individuals with hip and/or knee osteoarthritis (OA). The use of wearable activity trackers (WATs) has been shown to increase PA and improve other health outcomes but little is known concerning their effect on work ability. The objectives of this study were to examine the effect of self-monitoring PA with a WAT on work ability, PA and work productivity among individuals of working age with hip and/or knee OA.

**Methods:**

Individuals (*n* = 160) were included and cluster-randomized to a Supported Osteoarthritis Self-management Program (SOASP) with the addition of self-monitoring PA using a commercial WAT for 12 weeks (*n* = 86), or only the SOASP (*n* = 74). Primary outcome was self-reported work ability measured with the Work Ability Index (WAI) and secondary outcomes were self-reported PA measured with the International Physical Activity Questionnaire – Short Form (IPAQ-SF) and work productivity, measured with the Work Productivity and Activity Impairment scale: Osteoarthritis (WPAI:OA) at baseline and after 3, 6 and 12 months. Data was primarily analysed with linear mixed models.

**Results:**

Participants with data from baseline and at least one follow-up were included in the analyses (*n* = 124). Linear mixed models showed no statistically significant difference between groups regarding pattern of change in work ability or PA, from baseline to follow-ups. Also, neither group had a statistically significant difference in work ability between baseline and each follow-up.

**Conclusion:**

The SOASP together with self-monitoring PA with a WAT did not have any effect on the primary outcome variable work ability. Participants already at baseline had good work ability and were physically active, which could have reduced the possibility for improvements. Future interventions should target a population with lower work ability and PA-level.

**Trial registration:**

ClinicalTrials.gov, NCT03354091. Registered 15/11/2017.

## Background

Osteoarthritis (OA) is a common musculoskeletal disorder [1] affecting both individuals and the society at large [2, 3]. OA often leads to pain, disability and reduced quality of life [2, 4] which have been shown to affect work ability [5] and productivity [6] with presenteeism (reduced work capacity) being more common than absenteeism [7–9]. In a large population-based cohort study conducted in Sweden, individuals with knee OA had an almost two-folded risk of sick leave and 40–50% increased risk of disability pension in comparison with the general population [10]. The recommended non-surgical core treatment for management of hip and knee OA is education and exercise [11]. In Sweden, a Supported Osteoarthritis Self-management Program (SOASP) is recommended as the first-line treatment of patients with OA in the hip, knee and hand [12, 13]. The intervention in the SOASP includes at least two theoretical group sessions presenting information about OA, exercise, self-management and can be supplemented by individual supervised exercise therapy sessions.

Physical activity (PA) and exercise has been shown to reduce pain, improve physical function and health-related quality of life for individuals with lower limb OA [14, 15]. Previous research has also reported that exercise in the workplace [16] and walking programs [17] are effective in improving work ability and reducing work place limitations in individuals with hip and/or knee OA. Moderate or vigorous PA (MVPA) for at least 150 min per week is recommended to all adults by the World Health Organization (WHO) to reduce the risk of all-cause mortality, coronary heart disease, type 2 diabetes, depression and several other diseases [18]. However, a majority of individuals with hip and/or knee OA do not meet these PA recommendations [19–21].

A popular and effective method to increase PA (particularly walking) in healthy populations [22–24] as well as in populations with OA and other chronic conditions [25, 26] is the utilization of wearable activity trackers (WATs). WATs are often worn on the wrist and are paired with a smartphone, tablet or computer application (app). They can be used to self-monitor PA and increase long-term PA participation [22]. WATs utilize behavior change techniques (BCTs) such as goal-setting, self-monitoring and non-specific rewards that are effective in improving adherence to PA in short- and long-term [27]. WATs have also been shown to have beneficial effects on other outcomes such as mobility [24] and cardiometabolic health [25]. To our knowledge, there are no published studies examining the effect of WAT-usage on work ability and work productivity among individuals with hip and/or knee OA.

Thus, the primary objective in this study was to examine the effects of adding self-monitoring PA with a WAT to the SOASP on work ability and the secondary objectives PA and work productivity among individuals of working age with hip and/or knee OA compared to the SOASP only. We expected that the intervention group would improve their work ability, PA and work productivity compared to the control group.

## Methods

### Design

We did a two-armed cluster-randomized (C-RCT) study with allocation ratio 1:1, comparing the SOASP with the SOASP together with self-monitoring PA using a commercial WAT (Fitbit Flex 2) for 12 consecutive weeks as an add-on. The C-RCT is registered in clinical trials, 15/11/2017 (No: NCT03354091) [28]. After trial commencement, an additional method (Facebook) was used to recruit participants due to low recruitment rate. Participants PA-levels and adherence to using the WAT during the intervention has previously been described [29].

### Participants and recruitment

Eligible for recruitment in this study were individuals of working age in southern Sweden with hip and/or knee OA. The inclusion criteria were: working ≥50% (20 h. /week), aged between 18 and 67 years, being able to understand Swedish in speech and writing and able to participate in PA. They also had to have access to a smartphone, tablet or computer to use the Fitbit-app and be able to wear a WAT for 12 weeks. Potential participants in this study were approached in two different ways. Physiotherapists at 28 healthcare centers and physiotherapy clinics in southern Sweden were contacted in 2017–2018 and asked to inform individuals participating in the SOASP about the research project. The physiotherapists informed the first author EÖ about planned SOASPs (*n* = 114). The different healthcare centers offered SOASPs from twice yearly up to 10 times yearly and each SOASP had between 3 to 15 participants. Participants in the SOASPs were given oral and written information about the research project, inclusion/exclusion criteria and how to register. Interested individuals (*n* = 43) that met the criteria self-registered on the project’s website using an electronic identification (ID) service [30] and thereby giving their informed consent. In 2018, a Facebook advertisement was added to recruit additional participants. Individuals that were interested registered on the project’s website and took part of a SOASP offered within the research project. EÖ was responsible for the SOASPs held within this project (*n* = 9) that consisted of three theoretical sessions. An individual visit with a physiotherapist (EÖ) was also offered.

### Intervention

Participants in both groups (intervention/control) took part of the SOASP, which is described more extensively elsewhere [12, 13]. The SOASP offers first line treatment for patients with hip, knee and/or hand OA and is generally offered in primary health care. The SOASP consists of at least two theoretical group sessions. Participants are also offered an individual appointment with a physiotherapist and introduced to specific exercises based on their needs and goals. Some healthcare centers or physiotherapy clinics offer additional group sessions with other health care professionals such as occupational therapists or dieticians; and supervised group training, often for a limited period, e.g. two times a week for 6 weeks.

The intervention in this study comprised the SOASP together with self-monitoring PA using a commercial wrist-worn WAT (Fitbit Flex 2) for 12 consecutive weeks as an add-on. The Fitbit Flex 2 continually estimates steps taken, distance traveled, and time in different activity levels. The Fitbit is waterproof and can be worn during swimming and showering. The device is worn inside a rubber wristband and has five small LED-lights but no display. The measurements are transmitted via Bluetooth from the device to a smartphone, tablet or computer app, which in turn transfers the data to the Fitbit servers. All registered activity data can be viewed anytime on the app or on the Fitbit user portal.

Each participant in the intervention arm met with EÖ and received the Fitbit. They were aided in installing the Fitbit app and synchronizing the device to the participant’s app as well as connecting the participant’s Fitbit account to the study via the project’s website. The participants in the intervention group were asked to wear the Fitbit for 12 weeks, from morning until bedtime. They were also asked to monitor their activity by using the app once a day. Asking them to use the app once per day facilitated self-monitoring and allowed for synchronization of the data from the device to the app. During the 12-week period, there were no planned reminders but if participants had several (4-5) days without registered activity, EÖ contacted them by e-mail to ensure that there were no technical issues.

The default activity goal of 10,000 steps per day was changed to 7000 because we wanted it to be achievable for the participants. Previous research has also suggested that 7000 steps per day might be an accurate estimate for meeting the recommended 150 min per week of MVPA [31, 32]. The other default activity goals (*distance, calories burned and bouted active minutes*) in the app remained unchanged. Participants received feedback with a notification from the Fitbit when they reached their goals.

### Outcomes and measurements

The primary outcome in this study was self-reported work ability. The secondary outcomes were self-reported PA and self-reported work productivity. All outcomes were assessed at baseline and follow-up at three, 6 and 12 months with an online-survey that was sent to the participants’ e-mail.

Work ability was measured with Work Ability Index (WAI), a self-reported instrument containing questions about health, work demands and sick leave [33]. The index is calculated from seven questions and ranges from 7 to 49. The index from WAI can also be categorized in four different categories with a higher number indicating higher work ability; “poor” 7–27, “moderate” 28–36, “good” 37–43 and “excellent” 44–49 [34]. The WAI has shown acceptable predictive validity [35] and test-retest reliability [36]. Only the index value was considered in the analyses in this study.

PA was measured with the International Physical Activity Questionnaire - Short Form, (IPAQ-SF) [37]. IPAQ-SF is self-reported and comprises nine questions about time spent in high intensity, moderate intensity, walking or sitting in the last 7 days. The outcomes of IPAQ-SF are Metabolic Equivalent of Tasks (MET) minutes/week and PA category score (low, moderate or high). MET-minutes and PA category were calculated for each individual according to the IPAQ-SF protocol [38]. In this study, only MET-minutes were used in the analyses.

Work Productivity was measured with the self-reported Work Productivity and Activity Impairment scale – osteoarthritis (WPAI:OA) [39]. WPAI:OA yields four scores: *absenteeism* (work time missed), *presenteeism* (impairment at work/reduced on-the-job effectiveness), *work productivity loss* (overall work impairment/absenteeism plus presenteeism) and *activity impairment* [40]. The scores are expressed as impairment percentages with higher numbers indicating greater impairment and less productivity.

### Sample size

A sample size calculation was made using the primary outcome variable work ability measured with WAI to determine the required number of participants with a power of 80% and a two-tailed significance level of 0.05. Effect sizes (between group differences) for WAI were pragmatically based on SD on WAI from previous studies [5, 41] with the approximation of 0.45 SD as the minimal clinically important difference [42]. The sample size calculation yielded approximately 80 participants per group.

### Randomization

Each SOASP held at healthcare centers or within the project was seen as a cluster. Each cluster was randomly allocated to either control or intervention group. The recruitment period ran from October 2017 to May 2019. The participants were recruited from 116 SOASPs at healthcare centers and nine SOASPs conducted exclusively within the project. A randomization plan (1:1) was generated from randomization.com with seven blocks and 128 number of sealed envelopes. EÖ handled the randomization plan and EEH handled the sealed envelopes. EÖ received information about planned SOASPs and used the sealed envelopes to cluster-randomize the SOASPs. A total of 125 sealed envelopes were used, 63 SOASPs were randomized to control and 62 SOASPs were randomized to intervention. Neither participants nor authors were blinded after the allocation to control or intervention.

### Data analysis

Statistical analyses were made using statistical package IBM SPSS Statistics version 27 [43]. The proportion of participants at baseline in different categories according to WAI and IPAQ-SF are described. Descriptive outcome scores for baseline and follow-ups are calculated as mean (SD). Due to skewed distribution and as recommended in the manual, IPAQ-SF MET-minutes were also calculated as median (IQR) [38].

A linear mixed model [44] was conducted to examine the effect of group (intervention/control) on participants’ scores on the primary outcome work ability and the secondary outcomes PA and work productivity from baseline to three, 6 or 12 month follow-ups. A first-order autoregressive covariance structure used was used. We did not adjust for baseline values or cluster. Group and time were added as fixed factors. Interaction Group*Time was further added to assess the difference in pattern of change (effect) between the groups. The secondary outcome variables PA and work productivity were non-normally distributed and therefore log-transformed with lg10 prior to analysis to meet the assumptions of the linear mixed model.

Changes in the outcome measures from baseline to each follow up time was also computed, and compared between groups through Analysis of covariance (ANCOVA) with adjustment for the baseline scores [45]. Mean adjusted differences and 95% confidence intervals (CI) were calculated. Since the change/difference scores were approximately normally distributed also for the IPAQ-SF and WPAI:OA, raw change/difference scores were included in all ANCOVA models.

Due to differences in the distribution of men and women in the groups, the analyses were also performed with sex included as a potential confounder. Adjusting for sex did not have an impact on the results and sex was hence not included in the final analyses. We also conducted a stratified analysis to see if the effect of the intervention differed for participant with low/moderate respectively high/excellent baseline results on WAI.. No interaction was found, and the low power (few participants with WAI low/moderate) may have played a role in this.

## Results

**Fig. 1:**
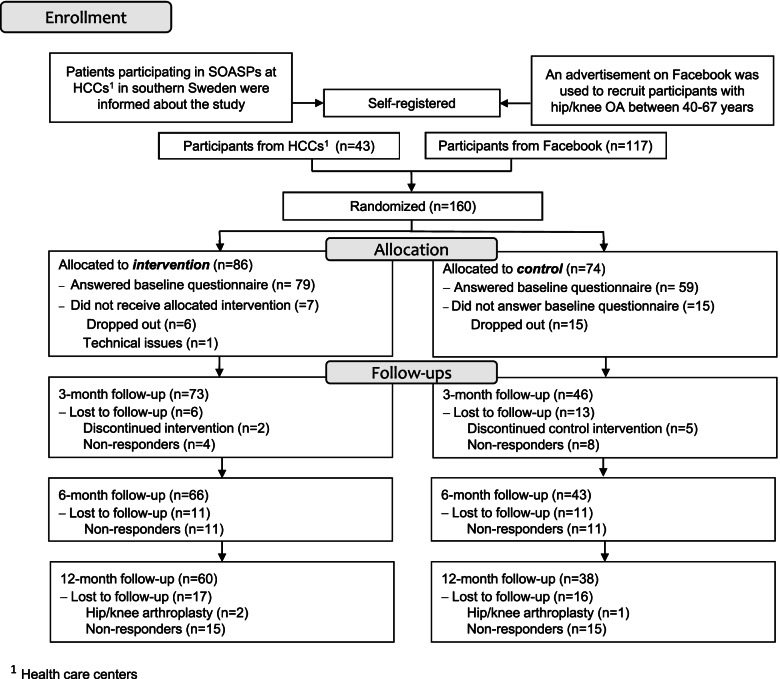
Flow of participants and the process of the study

We included individuals (*n* = 160) of working age with hip and/or knee OA from October 2017 until May 2019 with the last follow-up in May 2020. Participant recruitment ended when sufficient number of participants were included. A flow diagram is shown in Fig. 1. Twenty-one individuals dropped out due to different reasons before answering the baseline questionnaire.

Only participants that answered the questionnaire at baseline and at least one follow-up, 77.5% (*n* = 124), were included in the analyses, 103 women and 21 men and the intention-to-treat principle was used. Participants were on average 55.8 (SD 5.7) years and a majority reported having sedentary work and being less physically active compared to before they were diagnosed with OA (Table 1). Some minor differences were observed between the groups, especially regarding distribution of men and women with a higher proportion of men in the control group. Participants in the control group that were included after August 2018 (*n* = 27) were asked if they had used a private WAT during the study and 37% (*n* = 10) participants answered yes. A majority of the participants in both groups reported *good* or *excellent* work ability at baseline and were categorized as being moderate or highly physically active (Table 1).

**Table 1:** Participants’ baseline characteristics and data (WAI and IPAQ categories) (*n* = 124)

Group	Intervention (*n* = 74)	Control (*n* = 50)
**Age (years), mean (SD)**	56.7 (±5.3)	54.5 (±6.2)
**Sex, % (n)**		
Female	87.8 (65)	76.0 (38)
Male	7.2 (9)	24.0 (12)
**Married or living with partner, % (n)**	75.7 (56)	74.0 (37)
**Children in household - yes, % (n)**	27.0 (20)	26.0 (13)
**Most affected joint, % (n)**		
Hip	21.6 (16)	30 (15)
Knee	78.4 (58)	70 (35)
**Education (postsecondary**), **% (n)**	67.6 (50)	62.0 (31)
**Percentage of full time employment, % (n)**		
0–25%	2.7 (2)	n/a
26–50%	6.8 (5)	n/a
51–75%	10.8 (8)	6.0 (3)
76–100%	78.4 (58)	92.0 (46)
Unemployed	1.4 (1)	2.0 (1)
**Physically demanding work, % (n)**		
No	68.9 (51)	76.0 (38)
Yes, several times a week	9.5 (7)	12.0 (6)
Yes, daily	18.9 (14)	12.0 (6)
Missing	2.7 (2)	n/a
**Sedentary work, sitting > 50%, % (n)**	51.4 (38)	58.0 (29)
Missing	2.7 (2)	n/a
**Regular usage of a WAT during the last three months before the intervention, % (n)**	40.5 (30)	36.0 (18)
Missing	2.7 (2)	2.0 (1)
**Present physical activity level compared to before OA, % (n)**		
More physically active	10.8 (8)	14.0 (7)
Less physically active	55.4 (41)	50.0 (25)
Equally physically active	32.4 (24)	36.0 (18)
Missing	1.4 (1)	n/a
**WAI, categorical, % (n)**		
Poor (7–27 points)	4.1 (3)	2.0 (1)
Moderate (28–36)	18.9 (14)	20.0 (10)
Good (37–43)	39.2 (29)	26.0 (13)
Excellent (44–49)	33.8 (25)	44.0 (22)
Missing	4.1 (3)	8.0 (4)
**IPAQ, categorical, % (n)**		
Low	18.9 (14)	10.0 (5)
Moderate	29.7 (22)	42.0 (21)
High	41.9 (31)	38.0 (19)
Missing	9.5 (7)	10.0 (5)

*SD* Standard deviation, *WAT* Wearable activity tracker, *OA* Osteoarthritis, *WAI* Work Ability Index, *IPAQ* International Physical Activity Questionnaire

### Primary outcome (work ability)

The linear mixed model showed no statistically significant interaction (Group*Time) for the primary outcome work ability (*p* = 0.948). Also, there were no statistically significant main effect for group (*p* = 0.305) or time (*p* = 0.155). The ANCOVA showed no statistically significant between group differences regarding change in work ability for any of the periods (baseline to 3, 6 or 12 month follow-up) (Table 2).

**Table 2:** Primary and secondary outcomes. Change within and difference between the groups from baseline to follow-ups

Outcome	Intervention			Control			Between group differences	
	n^a^	Mean (SD)	Adj.* change from baseline [95% CI]	n^a^	Mean (SD)	Adj.* change from baseline [95% CI]	Adj.* difference [95% CI]	*p*
**WAI**								
Baseline	71	40.1 (6.3)	n/a	46	41.6 (6.8)	n/a	n/a	n/a
3-month follow-up	70	39.4 (7.3)	−0.6 [− 1.9, 0.6]	45	41.0 (6.9)	−0.8 [− 2.4, 0.8]	0.2 [− 1.8, 2.1]	*0.877*
6-month follow-up	62	39.1 (7.4)	−1.0 [− 2.1, 0.2]	40	41.2(6.2)	− 1.4 [− 2.8, 0.0]	0.4 [− 1.4, 2.2]	*0.650*
12-month follow-up	54	40.1 (6.4)	−0.6 [− 1.7, 0.6]	36	40.2 (7.3)	− 1.0 [− 2.5, 0.4]	0.5 [− 1.4, 2.3]	*0.618*
**IPAQ-SF MET-minutes/week**								
Baseline	67	3167 (2410)	n/a	45	2654 (1817)	n/a	n/a	n/a
3-month follow-up	64	3471 (2395)	647 [146, 1148]	41	2864 (1908.28)	−95 [− 698, 509]	741 [−44, 1526]	*0.064*
6-month follow-up	61	3319 (2527)	365 [−191, 921]	43	2918 (1809)	139 [− 530, 808]	226 [− 653, 1104]	*0.611*
12-month follow-up	57	2774 (2114)	−3 [− 511, 505]	37	2636 (1714)	− 136 [− 763, 491]	133 [− 679, 945]	*0.745*
**WPAI:OA absenteeism (%)**								
Baseline	62	2.4 (14.1)	n/a	44	0.0 (0.0)	n/a	n/a	n/a
3-month follow-up	56	1.2 (6.9)	−0.6 [−2.3, 1.0]	40	0.3 (2.2)	−1.1 [− 2.9, 0.7]	0.5 [− 2.0, 2.9]	*0.711*
6-month follow-up	49	3.1 (12.1)	1.1 [−1.8, 4.0]	32	0.7 (2.9)	−0.9 [−4.4, 2.7]	2.0 [−2.6, 6.6]	*0.390*
12-month follow-up	52	3.8 (13.7)	2.1 [−1.5, 5.6]	33	1.7 (8.7)	0.2 [−4.3, 4.6]	1.9 [−3.8, 7.6]	*0.508*
**WPAI:OA presenteeism (%)**								
Baseline	66	19.7 (25.7)	n/a	45	18.7 (24.9)	n/a	n/a	n/a
3-month follow-up	68	11.8 (20.2)	−8.1 **[−13.1, −3.1]**	43	19.8 (28.6)	3.1 [−3.1, 9.4]	−11.3 **[− 19.3, − 3.2]**	***0.006***
6-month follow-up	55	16.5 (24.6)	−2.8 [− 8.8, 3.2]	41	13.9 (22.9)	−3.2 [− 10.1, 3.7)	0.4 [− 8.7, 9.5]	*0.934*
12-month follow-up	53	14.2 (20.8)	−0.5 [−5.8, 4.9]	37	18.9 (25.4)	4.2 [−2.3, 10.7]	−4.6 [−13.1, 3.8]	*0.277*
Baseline	61	18.6 (24.9)	n/a	44	18.0 (24.7)	n/a	n/a	n/a
3-month follow-up	56	15.0 (22.5)	−6.1 **[−12.1, −0.2]**	40	18.9 (26.5)	3.2 [−3.5, 9.9]	−9.3 [**−18.3, − 0.4**]	***0.042***
6-month follow-up	49	19.5 (26.9)	0.4 [−5.8, 6.7]	32	12.6 (19.9)	0.1 [−7.6, 7.8]	0.3 [−9.7, 10.3]	*0.947*
12-month follow-up	52	16.3 (24.8)	2.1 [−2.9, 7.2]	32	18.8 (23.6)	5.0 [−1.3, 11.3]	−2.9 [− 10.9, 5.2]	*0.481*
**WPAI-OA activity impairment (%)**								
Baseline	74	30.9 (24.7)	n/a	50	28.8 (23.4)	n/a	n/a	n/a
3-month follow-up	71	26.6 (27.0)	−4.4 [−9.5, 0.7]	46	26.1 (25.9)	−3.7 [−10.0, 2.7]	−0.7 [−8.8, 7.5]	*0.868*
6-month follow-up	61	35.6 (31.4)	5.9 [−0.7, 12.4]	43	25.1 (25.4)	−3.0 [−10.8, 4.8]	8.8 [−1.4, 19.0]	*0.089*
12-month follow-up	60	29.0 (29.4)	0.7 [−4.9, 6.3]	38	22.9 (21.0)	−3.3 [−10.3, 3.8]	4.0 [−5.0, 13.0]	*0.382*

Analysis of Covariance (ANCOVA) were performed to analyze change within and difference between the groups

*SD* standard deviation, *CI* confidence interval, *WAI* Work Ability Index, *IPAQ-SF* International Physical Activity Questionnaire – Short Form, *MET* Metabolic Equivalent of Task, *WPAI:OA* Work Productivity and Activity Impairment scale: Osteoarthritis

^a^n denotes the number of participants with analyzable data for each variable/subscale

^*^Adjusted for baseline values

### Secondary outcomes (PA and work productivity)

**Fig. 2:**
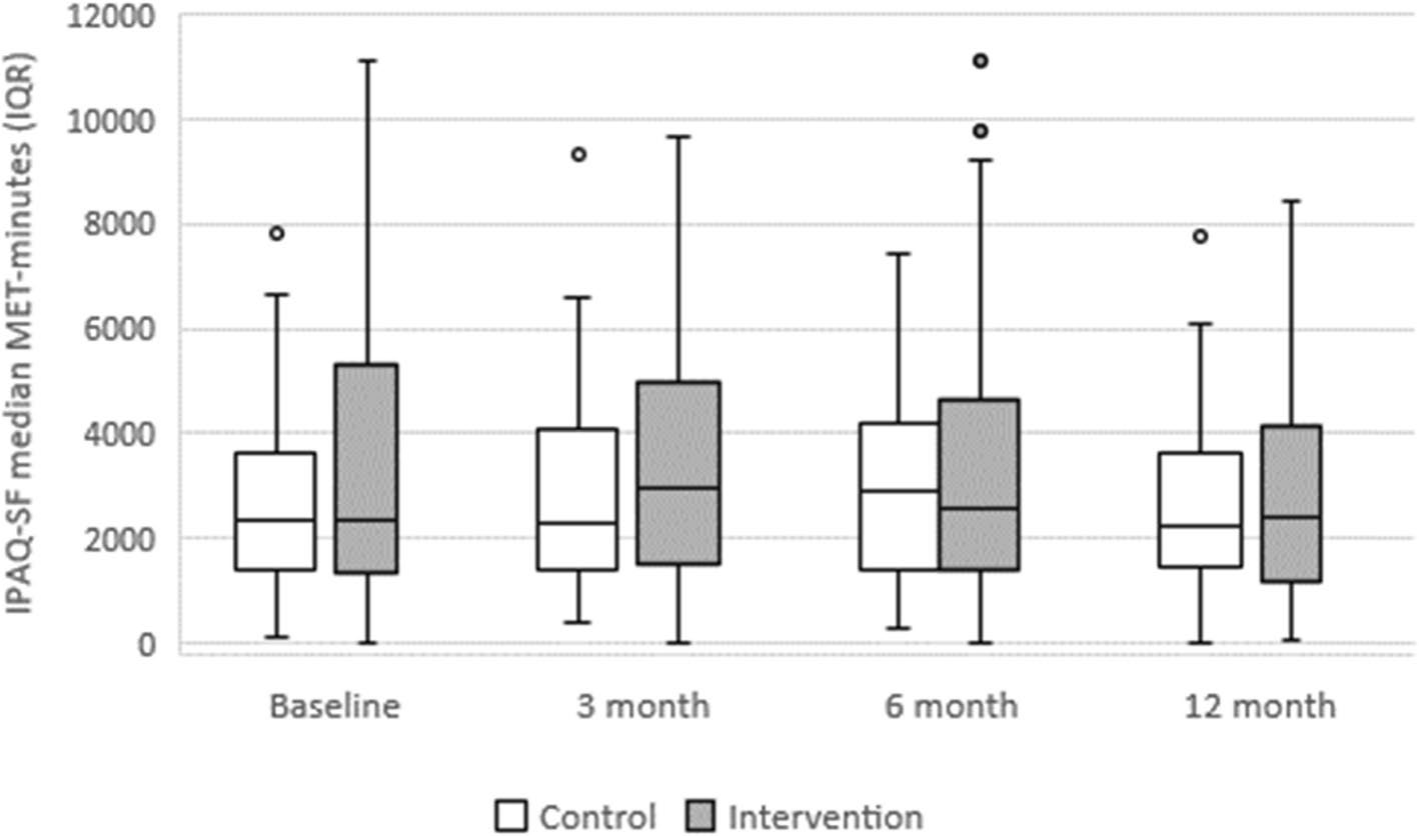
Median Metabolic Equivalent of Task (MET)-minutes based on International Physical Activity Questionnaire – Short Form (IPAQ-SF) for each group and measurement including interquartile range (IQR) and minimum-maximum range. The dots represent outliers

Median MET-minutes at baseline were 2335 (IQR 1408–3605) minutes for the control group and 2358 (IQR 1314–5310) minutes for the control group (Fig. 2). The linear mixed model showed no statistically significant interaction (Group*Time) for IPAQ-SF, WPAI:OA scores *absenteeism*, *work productivity loss and activity impairment*. There were, however, a statistically significant main effect for time for WPAI:OA *activity impairment*, *p* = 0.013 and a statistically significant interaction effect (Group*Time) for WPAI:OA *presenteeism*, *p* = 0.010.

ANCOVA with adjustment for baseline values, showed no statistically significant difference between or within groups regarding change in IPAQ-SF, WPAI:OA *absenteeism* and WPAI:OA *activity impairment,* from baseline to follow-ups. There were a statistically significant difference between the groups regarding change between baseline and 3 month follow-up for WPAI:OA *presenteeism* and WPAI:OA *work productivity loss* but no statistically significant difference between the groups regarding change from baseline to 6 or 12 month follow-up (Table 2).

## Discussion

In this C-RCT, we examined the effect of the SOASP with the addition of self-monitoring PA with a WAT compared to the SOASP alone on work ability, PA and work productivity in individuals with hip and/or knee OA of working age. Contrary to our expectation, we did not find any effect of the intervention on the primary outcome work ability, the secondary outcome PA and only to some minor extent work productivity (*absenteeism* and *activity impairment*).

The participants in this study had on average high scores on WAI throughout the study period, with both groups having a mean score ranging from 39.2 to 41.6, consequently falling within the “good” work ability classification on WAI [34]. Both control and intervention group had a lower mean WAI-score at 3 month (reduction with 1.4% respectively 1.7%) and 6 month follow-up (reduction with 1.0% respectively 2,5%) compared to baseline. However, the changes were small and not statistically significant. The scores on WAI in this study are comparable [5, 16] or higher [41] than the scores reported in previous studies on working OA-populations, indicating less possibility for improving the score on WAI.

Participants were on average also highly physically active already at baseline according to IPAQ-SF with a majority of the participants in PA-categories *moderate* and *high*. The intervention group had a statistically significant increase in mean MET-minutes from baseline to 3 months, which might indicate an effect of the intervention, but the difference in change between the groups was not statistically significant. The already high levels of PA at baseline might have limited the potential for improvement. According to a systematic review [46], lack of leisure-time vigorous PA, poor musculoskeletal capacity and high physical work load are among important factors associated with a poor work ability. In contrast, a majority of the participants in this study were moderately or highly physically active and did not have a physically demanding work which might explain why a majority of the participants in this study had good or excellent work ability.

The results on WPAI:OA showed a very low absence from work due to OA but a larger presenteeism/impairment at work. This result is in line with the results from previous research [7–9] reporting a higher prevalence of presenteeism than absenteeism. In addition, a systematic review reported that individuals with OA experience difficulties at work and have a lower work productivity but still remain at work [47]. Presenteeism/impairment at work decreased significantly from baseline to 3 month follow-up in the intervention group and there was a significant difference between the groups regarding change from baseline to the 3 month follow-up. However, this finding should be interpreted with caution since the difference did not persist throughout the remaining follow-ups.

Some limitations need to be considered. The first limitation regards selection bias. Participants self-registered to the study and a majority of them were recruited from the Facebook advertisement. This method of recruitment requires more effort from potential participants and consequently, we believe that many of the participants in this study already had an interest in e-health and were physically active. Also, using Facebook to recruit participants is a cost-effective and time saving method but leads to an over representation of young, white women [48]. In comparison with a large Swedish OA-cohort [49], the participant characteristics in this study differed on several points. In our study, participants had a lower mean age and there were higher proportions of females and participants with post-secondary education compared to the large cohort [49]. Some of these differences might be explained by the inclusion of only working age individuals in this study. However, we still believe that the participants in this study are probably not representative of the general population of working individuals with hip and/or knee OA. An additional limitation is also related to selection bias. Almost 40% of the participants reported already regularly using a WAT prior to taking part in this study, which supports the assumption that the participants in this study already were interested in e-health and monitoring PA. This corresponds well to the findings of a previous study reporting that WAT-use was associated with being female, below 60 years of age, having a post-secondary education and meeting PA guidelines [50]. Another limitation is that some of the participants in the control group used their own WAT during the study. Unfortunately, we only became aware of this after completion of the intervention. Lastly, there were a higher number of dropouts in the control group, particularly in an early stage before filling out baseline questionnaire. The reasons for dropping out were mainly a lack of time, a change of heart or changed conditions at work or family but we can not rule out that they dropped out because they did not receive the intervention. We do not believe that the dropouts changed the results in this study, but no dropout analysis were performed so we can only speculate on this.

Several systematic reviews with meta-analysis have established that WAT-use is effective to increase PA [22, 23, 26] which in turn might improve cardiometabolic health and mobility [24, 25]. In this study, we did not find any effect on the primary outcome variable work ability and only inconsistent results on the secondary outcome variables. In a previous study within this research project, we reported objective PA-data from the Fitbits worn by the intervention group in this RCT. In short, the participants had a high PA-level throughout the intervention, walking on average more than 10,000 steps per day and spending more than 300 min in MVPA/week although there was a slight decrease in PA during the 12 weeks [29]. This further strengthens our beliefs that the participants in our study already were highly physically active. They also had good work ability, low absenteeism and relatively low presenteeism, which probably leaves little room for improvement. We believe that the intervention in this study might have been more effective in a population with lower work ability, PA-level and work productivity. Future research should target individuals with hip and/or knee OA with low work ability and low PA-levels to examine if WAT-use could have an effect on work ability and other health outcomes in this population.

## Conclusion

The results in this C-RCT showed that the intervention, comprising the addition of self-monitoring PA with a WAT to the SOASP, did not have any effect on the primary outcome work ability compared to the SOASP alone. In general, the participants already had a good work ability and were physically active at baseline which might have reduced the possibility for improvements. Future research should target a population with low work ability and low PA-level.

## Data Availability

The datasets used and analysed during the current study are available from the corresponding author on reasonable request.

## Abbreviations

OA: Osteoarthritis
SOASP: Supported Osteoarthritis Self-management Program
PA: Physical activity
MVPA: Moderate and vigorous physical activity
WHO: World Health Organization
WAT: Wearable activity tracker
App: Application
BCT: Behavior change techniques
C-RCT: Cluster-randomized controlled trial
EÖ: Elin Östlind
WAI: Work Ability Index
IPAQ-SF: International Physical Activity Questionnaire – Short Form
WPAI:OA: Work Productivity and Activity Index:Osteoarthritis
MET: Metabolic Equivalent of Task
SD: Standard deviation
IQR: Interquartile range
ANCOVA: Analysis of covariance
